# Neurodegenerative Biomarkers in Populations with Intellectual Disabilities: Diagnostic and Therapeutic Capacities

**DOI:** 10.3390/ijms262412001

**Published:** 2025-12-13

**Authors:** Ainaz Shateri, Mohammad Tahan

**Affiliations:** 1 Department of Psychology, Florida International University, Miami, FL 33199, USA; ashat010@fiu.edu; 2 Department of Psychology, University of Tehran, Tehran P.O. Box 14155-6456, Iran; 3 American Psychological Association, Washington, DC 20002, USA

**Keywords:** biomarkers, neurodegenerative diseases, intellectual disability, early diagnosis, personalized treatment, Down syndrome

## Abstract

Populations with intellectual disabilities, especially individuals with genetic syndromes such as Down syndrome, are at very high risk of developing neurodegenerative diseases. This article aims to systematically review the capacities and limitations of biomarkers in the diagnosis and treatment of these diseases in these vulnerable populations. A narrative review was conducted using a systematic search of PubMed, Scopus, and Web of Science for studies published between 2000 and 2025 on biomarkers in intellectual disability and neurodegenerative diseases. Peer-reviewed articles in English or Persian were included, and the extracted data were synthesized thematically. Findings show that various biomarkers, including protein biomarkers (such as Aβ and tau), imaging (such as PET and MRI), genetic biomarkers, and fluid-based (blood and CSF) biomarkers, have significant potential in early diagnosis, monitoring disease progression, and evaluating treatment response. However, the use of these biomarkers in the population with intellectual disabilities faces unique challenges, including inherent biological heterogeneity, the presence of comorbidities, methodological barriers in assessment, and complex ethical considerations. The final conclusion indicates that achieving the maximum potential of these biomarkers requires the development of standardized and validated protocols for this specific population, conducting further longitudinal studies, and seriously considering ethical issues. This review emphasizes the importance of international collaborations and multidisciplinary approaches for transforming clinical care for these individuals.

## 1. Introduction

Individuals with intellectual disability, especially those who live with specific genetic syndromes, are at higher risk of developing neurodegenerative diseases. The increased risk of developing neurodegenerative diseases in individuals with intellectual disability is the result of the interaction of genetic, biological, environmental, and social factors [1]. Neurodegenerative diseases can cause the intensification of intellectual disability, the creation of cognitive disorders, and consequently a further reduction in the quality of life and daily functioning of individuals with intellectual disabilities [2].

Neurodegenerative diseases (nerve-destructive diseases) are a group of progressive disorders that are characterized by the gradual destruction of neurons and the loss of nervous functions. These diseases can cause the creation or intensification of intellectual disability in affected individuals [3]. Common neurodegenerative diseases include Alzheimer’s, Parkinson’s disease, frontotemporal degeneration, Huntington’s disease, and amyotrophic lateral sclerosis. These diseases, through different mechanisms such as the accumulation of abnormal proteins (like amyloid-beta in Alzheimer’s and alpha-synuclein in Parkinson’s), oxidative stress, neural inflammation, and genetic factors, cause the destruction of neurons [4]. When neurons are destroyed and die, this process leads to the occurrence of a wide range of symptoms that affect the different functions of the individual, including mental abilities, motor abilities, and even vital functions such as breathing and speech. The gradual decline of cognitive function is considered one of the common and prominent signs of all neurodegenerative diseases. Other common symptoms of these diseases include impairment in executive and mental functions, gradual loss of muscle control, the need for longer time to learn new skills, memory weakness, confusion and disorientation of place and time, restlessness and emotional instability, isolation and social withdrawal, perceptual hallucinations, thought delusions, depressed mood, and also the experience of unwanted and disturbing thoughts and feelings [5].

There are several neurodegenerative diseases that primarily manifest with motor dysfunction while cognitive functions remain largely intact, especially in the early or even throughout the disease course. For instance, amyotrophic lateral sclerosis (ALS) is characterized majorly by motor neuron degeneration and many patients retain cognitive function, although some show mild executive deficits [6,7].

Intellectual disability is directly associated with an increased risk of neurodegenerative diseases. For example, individuals with Down syndrome almost always show pathological signs similar to Alzheimer’s disease after the age of 20. This is due to the presence of the APP gene on chromosome 21, which leads to the overproduction of amyloid-beta protein and the formation of amyloid plaques in the brain [8]. Individuals with intellectual disability often experience higher levels of oxidative stress and neural inflammation, which can accelerate neuronal destruction [9]. Dysfunction in the antioxidant system and the accumulation of free radicals cause damage to proteins, lipids, and the DNA of nerve cells [10]. The accumulation of pathological proteins such as tau and alpha-synuclein, due to defects in the cellular clearance system, leads to neural destruction. This phenomenon is particularly evident in syndromes such as Down syndrome and fragile X syndrome [11]. Mutations in the polyglutamine-binding protein gene PQBP1 have a direct association with X-chromosome-linked intellectual disability (such as Renpenning syndrome). These mutations lead to the production of abnormal PQBP1 protein, which creates dysfunction in nerve performance [12]. Individuals with intellectual disability (especially those with Down syndrome) have an increased predisposition to developing neurodegenerative diseases [13].

The presence of unusual or altered clinical manifestations in these patients creates significant diagnostic and differential diagnostic challenges for specialists [13]. These diagnostic complexities arise from the overlap of baseline symptoms of intellectual disability with neurodegenerative signs, the presence of common comorbidities, and possible differences in the pathophysiology of the disease in this specific population [14,15].

Neurodegenerative (neurally destructive) diseases, are progressive disorders that increase in severity over time. At present, treatment of these diseases is mainly based on symptomatic control and delaying the process of disease progression, and the common approaches include pharmacotherapy, physiotherapy, occupational therapy, and palliative care. Although there is no definitive cure for these disorders [5]. But biomarkers, as objective and measurable indicators, have significant potential in improving the management and care of these diseases, especially in populations with intellectual disability. These biomarkers can help in early diagnosis, monitoring the progression of the disease, and evaluation of response to treatment. Types of biomarkers include: imaging biomarkers (MRI, PET, CT scan); cerebrospinal fluid biomarkers (amyloid-beta, tau); blood biomarkers (neurofilament light); and genetic biomarkers (genetic tests for specific mutations) [16].

The promise of biomarkers is supported by a growing body of international research. For instance, studies in Down syndrome populations have consistently shown that plasma biomarkers like Aβ42/40 and p-tau181, as well as CSF biomarkers and amyloid-PET imaging, are strongly associated with the onset and progression of Alzheimer’s disease [17,18,19,20]. Similarly, research on Fragile X syndrome has linked reduced FMRP protein and neuroinflammatory markers to underlying neurological dysfunction [21,22].

In the Iranian context, research has also contributed to this field, though often with a different focus. Important domestic studies have confirmed the vital role of biomarkers in diagnosing and treating neurodegenerative diseases like Alzheimer’s and Parkinson’s [23]. Furthermore, other studies have extensively investigated the genetic architecture of intellectual disability within the Iranian population, highlighting factors such as consanguineous marriages and identifying specific genetic causes like Fragile X syndrome and autosomal recessive microcephaly [24]. However, a notable research gap remains, as limited studies in Iran have specifically explored neurodegenerative biomarkers in populations with intellectual disability. This underscores the necessity for more targeted research in this area. In this regard, the use of biomarkers in the population with intellectual disability faces unique challenges [23]. The challenges of using biomarkers in the population with intellectual disability include multiple biological, methodological, and clinical factors. The inherent heterogeneity of this population in terms of genetic and environmental causes leads to diverse biomarker patterns that make the interpretation of results difficult. The presence of multiple common comorbidities and the use of various medications can affect biomarker levels and complicate the distinction between biomarkers specific to intellectual disability and factors related to comorbid diseases. Methodological limitations include problems in collecting invasive biological samples, the lack of gold-standard criteria, small sample sizes in studies, and heterogeneous research designs. Physiological differences in the immune and metabolic systems of these individuals also act as confounding factors. In addition, ethical challenges related to obtaining informed consent and the need to adapt standard protocols to the specific characteristics of this population are other important obstacles. The dynamic and varying patterns of biomarkers over time also make longitudinal monitoring and the evaluation of response to treatment difficult. Overcoming these challenges requires the development of personalized approaches, adaptive protocols, and longitudinal studies with sufficient sample sizes [25,26,27]. These challenges make the application of biomarkers in this population require personalized and specialized approaches. Therefore, this article, using reliable theoretical sources and focusing on the challenges of using biomarkers in the population with intellectual disability, systematically examines the capacities and limitations of these biomarkers in the diagnosis and treatment of neurodegenerative diseases. The ultimate goal is to provide a comprehensive review of the role of biomarkers and to outline future perspectives for improving the care of this vulnerable population.

## 2. Method

### 2.1. Study Design

This study was conducted as a Narrative literature Review, synthesizing findings from domestic and international research on biomarkers associated with neurodegenerative diseases in populations with intellectual disability. The review followed standard methodological recommendations for systematic reviews in biomedical sciences [28].

### 2.2. Search Strategy

A comprehensive search was performed across multiple databases, including PubMed, Scopus, Web of Science, and Google Scholar. The search covered studies published between 2000 and 2025 in order to capture both foundational and recent developments. The search strategy employed combinations of keywords and Boolean operators such as biomarkers or neurodegenerative biomarkers, intellectual disability or specific syndromes including Down syndrome, Fragile X, and Rett syndrome, as well as neurodegenerative conditions such as Alzheimer’s disease, Parkinson’s disease, and Huntington’s disease. Terms related to biomarker modalities, including cerebrospinal fluid, blood biomarkers, genetic biomarkers, and imaging biomarkers, were also included [23,29]. Reference lists of selected articles were further screened to identify additional relevant studies.

### 2.3. Inclusion and Exclusion Criteria

Studies were eligible for inclusion if they were peer-reviewed, published in English or Persian, and provided accessible full texts. Only original research articles and review papers that examined biomarkers in relation to neurodegenerative diseases among populations with intellectual disability were included. Studies were required to report diagnostic, prognostic, or therapeutic applications of biomarkers. Exclusion criteria applied to studies that were unrelated to neurodegenerative diseases, that addressed intellectual disability without examining biomarkers, or that were conference abstracts, editorials, or commentaries without primary data.

### 2.4. Data Extraction

For each eligible study, information was extracted on author names, year of publication, country of study, characteristics of the study population including sample size, age, and type of intellectual disability, the specific neurodegenerative disease examined, and the type of biomarker investigated. Biomarkers were categorized as genetic, molecular, imaging, fluid-based, electrophysiological, or metabolic. Key findings related to diagnostic, prognostic, and therapeutic applications were summarized [25,26]. The extracted data were organized into comparative tables to facilitate synthesis across biomarker types and syndromes.

### 2.5. Quality Assessment

Methodological quality was evaluated using established checklists for biomedical research. Criteria included adequacy of sample size, clarity and reproducibility of biomarker measurement methods, and relevance to populations with intellectual disability. Studies with insufficient methodological detail were retained for narrative synthesis but were interpreted with caution.

### 2.6. Data Synthesis

Findings were synthesized using a thematic analysis approach. Biomarkers were grouped into categories and further stratified by syndrome, with emphasis on Down syndrome, Fragile X syndrome, and Rett syndrome. Areas of agreement, contradictory findings, and gaps in research were identified. Differences between domestic studies conducted in Iran and international studies were given particular attention in order to contextualize disparities in methodology and application [30,31].

## 3. Results

### 3.1. Neurodegenerative Diseases: Epidemiology and Mechanisms

Neurodegenerative diseases are a group of progressive and disabling disorders of the central nervous system that are characterized by the gradual destruction and death of neurons in specific regions of the brain, leading to progressive cognitive, motor, and functional disorders. These diseases ultimately affect the individual’s independence and quality of life. The most common types include Alzheimer’s disease, Parkinson’s disease, frontotemporal degeneration, Huntington’s disease, and amyotrophic lateral sclerosis (ALS) [32].

Alzheimer’s disease is the most common cause of dementia in the elderly and accounts for about 60–70% of dementia cases. However, the neuropathological changes associated with AD, such as amyloid-β deposition and tau protein aggregation, can also be present in individuals without dementia. This condition, often termed preclinical Alzheimer’s disease, represents the earliest phase of the disease process before the onset of measurable cognitive decline. Large-scale biomarker and neuroimaging studies have shown that approximately 10–30% of cognitively normal adults aged 60–80 years exhibit amyloid-β positivity, indicating the presence of Alzheimer’s-related pathology despite the absence of clinical symptoms [33]. Its prevalence increases significantly with age and doubles every 5 years after the age of 65 [32]. Parkinson’s disease is the second most common neurodegenerative disease, affecting about 1–2% of individuals over the age of 60 [34]. Frontotemporal degeneration is the third most common cause of early-onset dementia and usually begins between the ages of 45 and 65 [35]. A systematic review and meta-analysis estimated the pooled prevalence of FTD at ≈9.17 per 100,000 population (95% CI: 3.59–23.42) which is quite high [36]. A systematic review and meta-analysis reported a global prevalence of approximately 4.88 per 100,000 individuals (95% CI: 3.38–7.06). However, the prevalence rates exhibit substantial regional variation. In North America and Europe, the prevalence ranges from 6 to 9 per 100,000, while in Asia, it is notably lower, around 0.4 to 1 per 100,000 [37]. A systematic review and meta-Analysis based on 124 studies reported a global crude ALS prevalence of 4.42 per 100,000 population (95% CI 3.92–4.96) and an incidence of 1.59 per 100,000 population (95% CI 1.39–1.81) [38].

Age is the most important risk factor for most neurodegenerative diseases. In addition, genetic factors including specific gene mutations and polymorphisms (such as the APOE ε4 allele for Alzheimer’s), as well as environmental and lifestyle factors such as diabetes, high blood pressure, obesity, physical inactivity, unhealthy diet and smoking, history of head trauma, and depression are also associated with an increased risk of these diseases. In Table 1, the epidemiology of common neurodegenerative diseases is shown.

Individuals with intellectual disability, especially those who have Down syndrome, are at very high risk of developing Alzheimer’s disease. People with intellectual disability are up to five times more likely than the general population to develop dementia [39]. In individuals with Down syndrome, Alzheimer’s disease appears in the fourth and fifth decades of life, and almost 90–100% of people in the seventh decade of life show the pathological features of Alzheimer’s. This increased risk is due to the presence of an extra copy of chromosome 21 in these individuals, which contains the gene for amyloid precursor protein (APP), leading to increased production and accumulation of amyloid-beta and increased risk of Alzheimer’s disease at relatively younger ages [13].

The pathophysiological mechanisms of neurodegenerative diseases are complex and multifactorial and include the accumulation of abnormal proteins, oxidative stress, neuroinflammation, and dysfunction in mitochondrial performance. In Alzheimer’s disease, the accumulation of amyloid-beta (Aβ) plaques and tau neurofibrillary tangles leads to neuronal destruction [40]. In Parkinson’s disease, the formation of Lewy bodies and the accumulation of alpha-synuclein cause the death of dopaminergic cells in the substantia nigra of the brain. TDP-43 and FUS proteins are also involved in other neurodegenerative diseases. In addition, the accumulation of oxidative stress caused by the production of reactive oxygen species (ROS) and the reduction in the antioxidant capacity of cells leads to mitochondrial damage and neuronal destruction. Microglial activation and neuroinflammation, through the secretion of pro-inflammatory cytokines, also contribute to the process of neuronal destruction [41].

Disruption in cellular metabolism and mitochondrial function, which leads to reduced cellular energy production, is also another contributing factor. Dysfunction in the autophagy system and the clearance of damaged proteins also causes the accumulation of abnormal proteins. From a molecular perspective, genetic and epigenetic mechanisms also play a significant role; specific genetic mutations (such as mutations in the APP, PSEN1, PSEN2 genes in familial Alzheimer’s [42] or the HTT gene in Huntington’s disease [43]) and epigenetic factors such as changes in DNA methylation can influence the expression of disease-related genes [44]. These pathological processes ultimately lead to impairment in synaptic communications and cell death [45].

Table 2 shows the pathological mechanisms in neurodegenerative diseases. These pathological processes ultimately lead to disruption in synaptic connections and cell death. Understanding these mechanisms is essential for developing new therapeutic strategies.

Fragile X syndrome, which is the most common inherited cause of intellectual disability, is associated with the expansion of CGG trinucleotides in the FMR1 gene, and studies have shown that these individuals may exhibit neuropathological changes similar to neurodegenerative diseases.

Environmental factors also play a role in the development of neurodegenerative diseases. Exposure to certain toxins, heavy metals, and pesticides has been associated with an increased risk of diseases such as Parkinson’s disease. In addition, some infectious agents, such as viruses, play a role in the onset of neurodegenerative diseases.

### 3.2. Intellectual Disability: Types and Association with Neurodegeneration

Intellectual disability is defined as a neurodevelopmental disorder characterized by significant limitations in intellectual functioning and adaptive behaviors, manifested during the developmental period. According to the Diagnostic and Statistical Manual of Mental Disorders (DSM-5), it is associated with an IQ below 70 and deficits in conceptual, social, and practical skills that appear before the age of 18 [46].

The bidirectional relationship between intellectual disability and neurodegenerative diseases can be examined from several perspectives. Individuals with intellectual disability, especially those with Down syndrome, are at higher risk of developing neurodegenerative diseases such as Alzheimer’s [47]. Studies show that up to 90% of individuals with Down syndrome after the age of 65 display pathological signs of Alzheimer’s, which is caused by trisomy of chromosome 21 and the overexpression of the amyloid precursor protein (APP) gene. On the other hand, neurodegenerative diseases can exacerbate existing intellectual disability or lead to new cognitive impairments in affected individuals [14].

The diagnosis of neurodegenerative diseases in the population with intellectual disability faces multiple obstacles, including the presence of baseline cognitive and functional deficits that make it difficult to distinguish between pre-existing symptoms and new disease manifestations, as well as the unusual presentations of neurodegenerative diseases in this population, which complicate clinical diagnosis [48].

Standard diagnostic tools designed for the general population often lack sufficient sensitivity and adequacy for individuals with intellectual disability, and conventional cognitive tests may not be applicable due to existing communication and cognitive limitations in this group [49].

The use of biomarkers in the population with intellectual disability requires special considerations, and although cerebrospinal fluid-based and blood-based biomarkers have been well validated in the general population, their effectiveness in individuals with intellectual disability may be limited due to biological heterogeneity and the presence of comorbid medical conditions [50].

Other challenges include limited access to specialized services, the need to adapt diagnostic protocols to the specific needs of these individuals, and the lack of robust research on the effectiveness of biomarkers in this population, which together lead to delays in timely diagnosis and treatment of neurodegenerative diseases in people with intellectual disability [48,51].

In terms of pathomechanisms, intellectual disability involves complex neurobiological mechanisms. In Down syndrome, the presence of three copies of chromosome 21 leads to overexpression of key genes, including APP and DYRK1A. Overproduction of the APP gene results in excessive generation of amyloid-beta peptides, whose accumulation facilitates the formation of amyloid plaques. These plaques, through the induction of oxidative stress, disruption of synaptic function, and triggering of neuronal apoptosis, increase neural vulnerability. On the other hand, overexpression of the DYRK1A gene contributes to mitochondrial dysfunction, abnormal phosphorylation of the tau protein, and the development of Alzheimer-like neuropathology [13].

In addition, shared inflammatory pathways exist between intellectual disability and neurodegenerative diseases. In disorders such as Down syndrome, chronic activation of microglia and the release of pro-inflammatory cytokines create a persistent inflammatory environment in the central nervous system. This chronic inflammation not only damages neurons but also weakens the blood–brain barrier, facilitating the entry of peripheral inflammatory factors into the central nervous system [52].

Metabolic disorders also play an important role in accelerating neural degeneration. In many inherited metabolic diseases associated with intellectual disability, defects in specific enzymes lead to the accumulation of toxic metabolites or to energy deficiency in neurons. These conditions not only cause neuronal death but also contribute to worsening cognitive and motor deficits [53]. Overall, the complex interaction between genetic, inflammatory, and metabolic factors creates a vicious cycle in which neural vulnerability increases and neurodegeneration accelerates. These processes are common not only in intellectual disability but also across many neurodegenerative diseases [25]. In Table 3, neurodegenerative diseases and the clinical features associated with intellectual disability are described.

### 3.3. Biomarkers

Biomarkers, as objective and measurable indicators, play an increasing role in the understanding, diagnosis, and management of neurological diseases. These biomarkers enable early detection, monitoring of disease progression, and evaluation of treatment response. In the context of neurological diseases, biomarkers are particularly important in complex disorders such as Alzheimer’s, Parkinson’s, multiple sclerosis, and rare neurological diseases [28].

Molecular biomarkers include a wide range of biological substances such as proteins, genes, and metabolites. In neurological diseases, notable examples include phosphorylated tau protein and amyloid-beta in Alzheimer’s, alpha-synuclein in Parkinson’s, and neurofilament light (NfL) in multiple sclerosis. These biomarkers can be measured in cerebrospinal fluid, blood, or peripheral tissues [14].

Imaging biomarkers are obtained using advanced techniques such as MRI, PET, and CT scan. These biomarkers allow for the assessment of structural and functional changes in the brain. For example, in Alzheimer’s disease, amyloid PET can reveal amyloid plaque accumulation, while in multiple sclerosis, MRI can identify hyperintense lesions [54].

Genetic and epigenetic biomarkers identify changes in DNA sequences or gene expression patterns. In rare neurological diseases such as hereditary sensory neuropathy (HSN) and Charcot-Marie-Tooth disease (CMT), specific gene mutations and microRNAs are used as diagnostic and prognostic biomarkers [55].

The role of biomarkers in neurological diseases is multidimensional. These biomarkers are used in early diagnosis, differentiation between disease subgroups, monitoring disease progression, evaluating treatment response, and predicting disease course. For example, in multiple sclerosis, serum NfL levels can indicate disease activity and response to treatment [56]. In the context of therapy, biomarkers serve as criteria for selecting patients for targeted treatments and for monitoring treatment effectiveness. In clinical trials, these biomarkers can be used as surrogate endpoints and can accelerate the process of developing new drugs [57,58].

Current challenges in the field of neurological biomarkers include standardization of measurement methods, validation in different populations, and integration of multimodal data. In addition, for rare neurological diseases, collecting sufficient samples for validation studies is considered a major challenge [23].

Biomarkers play a vital role as diagnostic, prognostic, and therapeutic monitoring tools in neurodegenerative diseases. These biomarkers enable early identification of disease, follow-up of progression, and evaluation of treatment response. Below is a comprehensive categorization of types of biomarkers in neurodegenerative diseases:

1. Protein biomarkers: Protein biomarkers are among the most important diagnostic indicators in neurodegenerative diseases:
  - Amyloid-beta (Aβ): The accumulation of this protein in the brain forms amyloid plaques, which are pathological features of Alzheimer’s disease. Measurement of the Aβ42/Aβ40 ratio in cerebrospinal fluid (CSF) and blood is used to diagnose Alzheimer’s.
  - Tau: Phosphorylated tau protein (p-tau) and total tau (t-tau) in CSF are used as biomarkers of neurodegeneration in Alzheimer’s disease. Increased levels of p-tau181 and p-tau217 in blood are also associated with disease progression.
  - Alpha-synuclein (α-syn): This protein is the main biomarker of Parkinson’s disease and dementia with Lewy bodies. Measurement of α-syn in CSF and blood is used for diagnosis and monitoring of these diseases [29,59]. A summary of key protein biomarkers, including Amyloid-beta, Tau, and Alpha-synuclein, is provided in Table 4.
2. Genetic and epigenetic biomarkers: These markers help identify genetic susceptibility and molecular mechanisms of disease:
  - Specific gene mutations: Mutations in the APP, PSEN1, and PSEN2 genes in familial Alzheimer’s and mutations in the LRRK2 and GBA genes in Parkinson’s are among the important genetic biomarkers [23].
  - Polymorphisms: The APOE ε4 variant is considered the strongest genetic risk factor for sporadic Alzheimer’s [29].
  - Epigenetic changes: Alterations in DNA methylation patterns and histone-modifying enzymes play roles in regulating the expression of disease-related genes [60]. Table 5 outlines the role of genetic mutations, polymorphisms, and epigenetic changes as biomarkers for neurodegenerative diseases.
3. Imaging biomarkers: Advanced imaging techniques enable direct visualization of pathological changes in the brain:
  - Amyloid PET imaging: The use of ligands such as Pittsburgh compound B (PiB) to identify amyloid plaques in Alzheimer’s disease.
  - Tau PET imaging: Ligands such as flortaucipir are used to identify tau neurofibrillary tangles [29].
  - Structural and functional MRI: Assessment of hippocampal atrophy, cortical volume loss, and connectivity changes in brain networks [61]. The technical specifications and comparison of these imaging biomarkers are provided in Table 6.
4. Body fluid biomarkers: These markers can be measured in different biological samples:
  - Cerebrospinal fluid (CSF):
    ○ Core Alzheimer’s markers: Aβ42/Aβ40 ratio, p-tau, t-tau [29,59].
    ○ Inflammatory markers: Inflammatory cytokines such as IL-6, TNF-α.
    ○ Neurofilament light (NfL): A marker of axonal degeneration [29].
  - Blood:
    ○ Blood-based biomarkers: Potential use of plasma-based biomarkers for non-invasive diagnosis.
    ○ Exosomes: Extracellular vesicles containing proteins and nucleic acids related to disease.
  - Other fluids:
    ○ Saliva: Measurement of α-syn in Parkinson’s and Aβ in Alzheimer’s.
    ○ Urine: Identification of metabolites related to oxidative stress [29].
  Body fluid biomarkers in neurodegenerative diseases are presented in Table 7.
5. Emerging biomarkers
  Recent advances have led to the identification of new biomarkers:
  - Long non-coding RNAs (lncRNAs): These play a role in regulating gene expression and may be used as diagnostic biomarkers [23].
  - Gut microbiome: Changes in the composition of the gut microbiome and the gut–brain axis in the pathogenesis of neurodegenerative diseases.
  - Inflammatory markers: Inflammatory cytokines and chemokines that play roles in neuroinflammatory processes [29].
  - Neuron-derived exosomes: Extracellular vesicles derived from neurons that contain pathogenic proteins such as Aβ and tau.

Biomarkers and their applications in neurodegenerative diseases are shown in Table 8.

### 3.4. Types of Biomarkers in Intellectual Disabilities

Intellectual disabilities are associated with a wide range of biomarkers that show distinct patterns in different syndromes. In Down syndrome, trisomy of chromosome 21 leads to the overproduction of the APP gene and causes increased levels of amyloid-beta peptides and phosphorylated tau protein, which ultimately facilitates the formation of amyloid plaques and neurofibrillary tangles. These biochemical changes are associated with reduced hippocampal volume, cerebellar hypoplasia, and impaired synaptic function, as well as abnormal EEG patterns including slow waves and impaired event-related potentials. In addition, increased inflammatory markers such as TNF-α, IL-1β, IL-6 and oxidative stress caused by elevated SOD1 play a role in the pathophysiology of this syndrome [62].

In Fragile X syndrome, expansion of the CGG trinucleotide in the FMR1 gene to more than 200 repeats leads to reduced or absent production of the FMRP protein, which plays a key role in regulating the translation of proteins involved in dendritic development and synaptogenesis. Reduced FMRP is associated with increased MMP-9 in cerebrospinal fluid, changes in neurotransmitter levels, and disruption of mGluR5 signaling pathways. Brain imaging shows enlargement of ventricles and abnormalities in the corpus callosum, while electrophysiology records increased sensitivity to sound and impaired auditory processing. Changes in the inflammatory cytokine profile such as IL-6 and TNF-α are also other features of this syndrome [63].

Rett syndrome is caused by mutations in the MECP2 gene, which lead to disruption of DNA methylation and reduced levels of brain-derived neurotrophic factor (BDNF). These changes are associated with increased glutamate levels and disturbances in GABA levels in cerebrospinal fluid, producing characteristic EEG patterns with sharp and slow waves. Brain imaging shows reduced brain growth and microcephaly, while metabolic disturbances include elevated ammonium and lactate in blood. Increased inflammatory cytokines and astrocyte activation are also prominent features of this syndrome [64].

Blood biomarkers play an important role in monitoring and prognosis in these syndromes. In Down syndrome, increased interleukin-6 and SOD1; in Fragile X syndrome, elevated FMR1 mRNA and cytokine changes; and in Rett syndrome, increased ammonium and metabolic profile disturbances are observed. These biomarkers are useful not only in diagnosis but also in tracking response to therapeutic interventions [65].

Imaging biomarkers provide valuable information about structural changes in the brain. Cerebellar hypoplasia and reduced hippocampal volume in Down syndrome, enlargement of brain ventricles in Fragile X syndrome, and reduced brain growth in Rett syndrome are among these findings. These changes are directly associated with cognitive and behavioral impairments in patients [66].

Electrophysiological patterns are also unique to each syndrome. Slow EEG waves in Down syndrome, auditory processing deficits in Fragile X syndrome, and sharp and slow wave patterns in Rett syndrome are applicable not only in diagnosis but also in evaluating disease progression and treatment response [67].

Metabolic and inflammatory biomarkers indicate the pathophysiological status of these disorders. Disturbances in folate metabolism and increased oxidative stress in Down syndrome, disruption of the mGluR5 pathway in Fragile X syndrome, and abnormalities in creatine metabolism in Rett syndrome are among these markers. Increased inflammatory biomarkers are also observed in all three syndromes, playing an important role in neurodegeneration [68].

Neurodevelopmental biomarkers such as delayed myelination, impaired dendritic growth, and reduced synaptogenesis indicate the impact of these syndromes on the growth and development of the nervous system. These changes progress over time and are directly correlated with the severity of clinical symptoms [53].

Finally, integrating information derived from these biomarkers can lead to the development of personalized approaches in the diagnosis and treatment of intellectual disabilities. The use of advanced techniques such as omics and artificial intelligence in analyzing these data can help identify complex patterns and foster the development of more effective interventions [13]. In Table 9, the biomarkers in intellectual disabilities are presented.

### 3.5. Diagnostic and Therapeutic Applications of Biomarkers in Intellectual Disabilities

Biomarkers play an increasing role in the diagnosis and management of neurodegenerative diseases and provide new opportunities for early intervention and targeted therapy. The diagnostic and therapeutic applications of these biomarkers can be examined in four main domains:

First, in the domain of early and differential diagnosis, biomarkers enable the identification of disease years before the appearance of clinical symptoms. For example, in Alzheimer’s disease, measurement of the Aβ42/Aβ40 ratio in cerebrospinal fluid and blood can detect amyloid deposition years before clinical symptoms emerge. In addition, phosphorylated tau (p-tau) is used as a more specific biomarker for tracking tau pathology. Studies have shown that a combination of different biomarkers (such as amyloid-PET along with measurement of p-tau in cerebrospinal fluid) can significantly increase diagnostic accuracy and enable differentiation between various subgroups of neurodegenerative diseases [69,70].

Second, in the field of prognosis and monitoring disease progression, biomarkers provide valuable tools for predicting disease course and treatment response. Neurofilament light (NfL), as a nonspecific marker of axonal degeneration, shows strong correlation with the speed of disease progression in multiple sclerosis and Alzheimer’s disease. Longitudinal studies have shown that elevated plasma NfL levels can predict the transition from mild cognitive impairment to full dementia. In addition, inflammatory markers such as high-sensitivity C-reactive protein (hs-CRP) and various interleukins can assist in monitoring disease activity and treatment response [71].

Third, in the domain of monitoring treatment response, biomarkers provide the ability to objectively evaluate the effectiveness of pharmacological and non-pharmacological therapies. In clinical trials, these biomarkers are used as surrogate endpoints and allow for faster assessment of therapeutic effects. For example, in Alzheimer’s disease, a reduction in p-tau levels in response to anti-amyloid therapies can be used as an indicator of treatment effectiveness. Similarly, in Parkinson’s disease, cerebrospinal fluid alpha-synuclein can monitor the response to disease-modifying therapies [14].

Fourth, in the field of new therapeutic targets, biomarkers have been considered not only as diagnostic tools but also as direct therapeutic targets. A better understanding of the pathophysiology of neurodegenerative diseases through the study of biomarkers has enabled the identification of new targets for the development of innovative therapies. For example, recent studies have focused on tau modulators and amyloid aggregation inhibitors. In addition, inflammatory biomarkers such as pro-inflammatory cytokines are being investigated as new targets for immunomodulatory therapies in neurodegenerative diseases [72].

Moreover, recent technological advances in ultra-sensitive assays (such as SIMOA technology) have made it possible to measure biomarkers at extremely low concentrations. These advances are particularly important for blood-based biomarkers, as their measurement in a non-invasive way allows longitudinal monitoring of disease in large populations [73].

The development of multiplex biomarker panels that combine pathological, inflammatory, and neurodegenerative markers has enabled more precise patient stratification and prediction of treatment response. This approach, moving toward personalized medicine, is considered the most promising research field in neurodegenerative diseases [72]. Overall, biomarkers have created a transformation in the management of neurodegenerative diseases and have become essential tools in clinical research and clinical applications. With continued progress in this field, it is expected that these biomarkers will play a very important role in prevention, diagnosis, and treatment of neurodegenerative diseases [74].

Biomarkers play a vital role in the diagnosis and management of intellectual disabilities and make possible the early identification and targeted intervention of disorders. In the domain of diagnosis, these biomarkers allow the detection of disorders at early stages. For example, in Down syndrome, trisomy of chromosome 21 and elevated APP levels are used as genetic and molecular biomarkers [62].

In Fragile X syndrome, expansion of CGG trinucleotides in the FMR1 gene and reduced FMRP protein are used as valid diagnostic biomarkers. These biomarkers are also useful in evaluating response to therapeutic interventions and predicting disease progression [63].

Inflammatory and immunological biomarkers also play an important role in the diagnosis and monitoring of intellectual disabilities. Elevated levels of inflammatory cytokines such as IL-6, IL-1β, and TNF-α in blood and cerebrospinal fluid have been reported in patients with neurodevelopmental disorders [64].

In the therapeutic domain, biomarkers allow monitoring of treatment response and assessment of intervention effectiveness. In clinical trials, these biomarkers are used as surrogate endpoints and provide faster evaluation of therapeutic effects [65].

Metabolic biomarkers also have applications in the management of intellectual disabilities. In some inherited metabolic disorders, measurement of specific metabolites in blood or urine can help monitor treatment response and adjust drug dosages [68].

The development of multiplex biomarker panels that combine genetic, biochemical, and inflammatory markers has enabled more precise patient stratification and prediction of treatment response. This approach moves toward personalized medicine [53].

Overall, biomarkers have created a transformation in the management of intellectual disabilities and have become essential tools in clinical research and clinical practice. With continued advances in this field, it is expected that these biomarkers will play a very important role in improving the diagnosis and treatment of intellectual disabilities [13].

### 3.6. Challenges and Limitations of Neurodegenerative Biomarkers in Populations with Intellectual Disability

The use of biomarkers in populations with intellectual disability faces unique challenges and limitations. Among the methodological challenges is the lack of sufficient validation of diagnostic tests for this specific population. Many biomarker measurement methods have been designed for the general population and may lack adequate accuracy and reliability in individuals with intellectual disability. In addition, comorbid medical conditions and the use of multiple medications can affect biomarker levels and distort results [69].

From a clinical perspective, the significant heterogeneity in the causes and manifestations of intellectual disability complicates the interpretation of biomarker results. Individual differences in baseline biomarker levels and the impact of pleiotropy in genetic syndromes make it difficult to establish cut-off values. Furthermore, many individuals with intellectual disability are unable to cooperate in performing invasive tests such as cerebrospinal fluid sampling [14].

Ethical considerations are of particular importance in this population. Obtaining informed consent from individuals who may have limited decision-making capacity requires special protocols and the involvement of legal representatives. There are also concerns regarding the potential for discrimination based on genetic information and the impact of test results on insurance and employment rights [49].

Economic and access-related limitations are also more pronounced in this population. The high costs of developing and implementing specialized biomarker tests, combined with the need to adapt protocols to the specific needs of these individuals, lead to significant increases in expenses. In addition, unequal access to specialized services and a shortage of well-equipped centers hinder the widespread use of these technologies in underserved populations [75].

To overcome these challenges, the development of standardized protocols tailored for populations with intellectual disability, the implementation of large-scale validation studies, the drafting of comprehensive ethical guidelines, and the creation of supportive systems to improve access are essential. Furthermore, investment in the development of non-invasive and cost-effective methods can help expand the use of biomarkers in this population [76].

## 4. Discussion

Our review highlights both significant alignments and critical disparities between domestic and international research on neurodegenerative biomarkers in intellectual disability populations. A key finding is the consistent emphasis on the diagnostic and prognostic utility of biomarkers across studies. For example, the domestic study by Shahverdi et al. [23] aligns with international consensus by demonstrating the high accuracy of CSF biomarkers and PET imaging for early and differential diagnosis of Alzheimer’s. This is corroborated by international studies, such as Hartley et al. [19], who found elevated amyloid-beta in adults with Down syndrome prior to clinical symptom onset.

However, a stark imbalance exists in the scope and focus of research. While international consortia have conducted extensive longitudinal studies, validating a range of fluid and imaging biomarkers for conditions like Down syndrome [18,20,30,77], domestic research in Iran has predominantly focused on elucidating the genetic causes of intellectual disability [24]. This focus is crucial, given the role of consanguinity in the population, but it has left a significant gap in the validation of neurodegenerative biomarkers specifically within the Iranian context. The limited number of domestic studies in this niche means that the promising blood-based biomarkers (e.g., Aβ42/40, p-tau181) emphasized in international literature [30] lack robust validation for the Iranian population.

The contradictions and disparities observed in the broader literature, such as the association between lower premorbid cognitive function and higher Alzheimer’s pathology [25,39], can be attributed to several factors beyond mere methodological differences. Population genetics likely play a role; the unique genetic background and higher rates of consanguinity in Iran may influence both the presentation of intellectual disability and the trajectory of neurodegeneration. Cultural and socioeconomic factors could affect access to specialized care, age at diagnosis, and exposure to environmental risk factors, all of which can confound biomarker levels and disease progression. Furthermore, the small sample sizes common in domestic studies on rare syndromes limit statistical power and generalizability. Therefore, the observed research gap is not merely a quantitative lack of studies but also a qualitative one, stemming from differences in population characteristics, research infrastructure, and historical research priorities.

In the context of Fragile X syndrome, studies such as Winarni and et al. [22] have linked reduced FMRP protein and increased inflammatory biomarkers to neurological disorders. This finding is consistent with the study by Akbari-Mobarak and et al. [24], which emphasized the importance of genetic factors in intellectual disability. Both studies point to the role of molecular mechanisms in the pathophysiology of intellectual disability. However, a major imbalance can be observed between domestic and international studies. While extensive international research has been conducted on biomarkers in Down syndrome and other disorders related to intellectual disability, domestic studies have mostly focused on genetic causes and have addressed neurodegenerative aspects to a much lesser degree. This research gap highlights the necessity of conducting more studies in Iranian populations.

Contradictions are also found in study methodologies. Some studies, such as Leverenz [18], have combined cerebrospinal fluid biomarkers with PET imaging, while others, like Strydom and et al. [77], have focused on genetic and inflammatory risk factors. This difference in methodology can lead to varying and sometimes contradictory results.

In conclusion, although significant progress has been made in identifying neurodegenerative biomarkers, the contradictions observed in studies underscore the need for standardized methodologies, attention to population differences, and longitudinal studies with sufficient sample sizes. Furthermore, international and multidisciplinary collaborations can help reduce these inconsistencies and advance the field.

## 5. Conclusions

By systematically reviewing the existing studies, it becomes clear that although neurodegenerative biomarkers hold enormous potential to revolutionize early diagnosis, disease monitoring, and personalized treatment of neurodegenerative disorders in populations with intellectual disability, realizing this potential requires overcoming the unique challenges of this field.

The first and most essential step is the design and implementation of long-term longitudinal studies with sufficient sample sizes that track the temporal trajectory of biomarker changes from childhood to adulthood across different syndromes (such as Down, Fragile X, and Rett syndrome). Such studies are necessary to establish baseline values and population-specific cut-off points that differ from those of the general population [13,28].

Second, future research must focus on the discovery and validation of novel biomarkers tailored specifically to this population. Given the intrinsic heterogeneity and distinct pathomechanisms of each syndrome, conventional biomarkers developed for the general population may lack sufficient accuracy and sensitivity. Therefore, investment in inflammatory biomarkers (such as specific cytokines), oxidative stress markers, and biomarkers related to synaptic dysfunction—reflecting the pathogenic mechanisms of these conditions—appears essential [14,39].

The third key direction is the development and standardization of unified methodological protocols for sample collection, processing, measurement, and data interpretation. Such standardization, which must also include the adaptation of cognitive and behavioral assessment methods to the characteristics of this population, is a prerequisite for cross-study comparability and for increasing diagnostic power [30].

Fourth, the future of this field depends on multi-modal data integration. Combining data derived from blood-based biomarkers, advanced imaging, genetics, and clinical assessments—leveraging artificial intelligence and machine learning algorithms—can lead to the development of stronger and more accurate predictive models for early diagnosis and personalized monitoring of disease [31].

Finally, serious attention to ethical considerations and the development of protective clinical guidelines is indispensable. Informed consent, confidentiality of genetic and sensitive data, and the prevention of stigmatization and discrimination require the establishment of robust ethical frameworks with the active involvement of individuals with intellectual disability, their families, and their representatives [49].

Achieving these ambitious goals will only be possible through international collaboration and the formation of strong research consortia so that through data and resource sharing, the challenge of small sample sizes in each syndrome can be overcome, ultimately transforming clinical care for this vulnerable population. 

## Figures and Tables

**Table 1:** Epidemiology of Common Neurodegenerative Diseases.

Disease	Prevalence	Age of Onset	Risk Factors
**Alzheimer’s disease**	60–70% of dementia cases 10–30% people without dementia	5–60 years	Age, genetic (APOE ε4), family history
**Parkinson’s disease**	1–2% of individuals over 60 years	50–70 years	Age, genetic and environmental factors
**Frontotemporal degeneration**	Third most common cause of early dementia 9.17 per 100,000 people	45–65 years	Genetic, family history
**Huntington’s disease**	4.88 in per 100,000 people	30–50 years	Genetic (HTT mutation)
**ALS (Amyotrophic lateral sclerosis)**	4.42 per 100,000 people	40–70 years	Age, genetic and environmental factors

**Table 2:** Pathophysiological Mechanisms in Neurodegenerative Diseases.

Main Mechanism	Disease	Related Proteins	Key Processes
**Protein aggregation**	Alzheimer’s, Parkinson’s	Tau, alpha-synuclein, Aβ	Formation of amyloid-beta plaques and neurofibrillary tangles
**Oxidative stress**	All neurodegenerative diseases	–	ROS production; mitochondrial damage
**Neuroinflammation**	Alzheimer’s, Parkinson’s, ALS	TNF-α, IL-1β, IL-6	Activation of microglia; secretion of cytokines
**Mitochondrial dysfunction**	Huntington’s disease	–	ATP production disorder; mitochondrial dysfunction
**Autophagy dysfunction**	Alzheimer’s, Parkinson’s, FTD	–	Accumulation of damaged proteins
**Genetic factors**	Familial Alzheimer’s, Huntington’s disease	APP, PSEN1, PSEN2, HTT	Genetic mutations, polymorphisms

**Table 3:** Main Types of Neurodegenerative Diseases and Clinical Features Associated with Intellectual Disability.

Disease	Pathological Features	Clinical Manifestations Related to Intellectual Disability
**Alzheimer’s disease**	Amyloid-beta plaques, neurofibrillary tangles	Memory impairment, language problems, cognitive disturbances
**Parkinson’s disease**	Lewy bodies, alpha-synuclein accumulation	Motor problems, cognitive impairment, depression disorders
**Huntington’s disease**	Huntington gene mutation	Cognitive disorders, behavioral changes
**ALS (Amyotrophic Lateral Sclerosis)**	Degeneration of motor neurons	Muscle weakness, speech disorders, respiratory problems

**Table 4:** Protein and Epigenetic Biomarkers.

Source Type	Biomarkers	Advantages	Limitations
Protein	Amyloid-beta (Aβ)	Detectable in CSF and blood; used to diagnose Alzheimer’s disease	May not differentiate between AD and other dementias; influenced by age and comorbidities
Protein	Tau (p-tau, t-tau)	Reflects neurodegeneration; p-tau181 and p-tau217 indicate disease progression	Levels may vary across individuals; requires standardized assays
Protein	Alpha-synuclein (α-syn)	Main biomarker for Parkinson’s disease and dementia with Lewy bodies; measurable in CSF and blood	Overlaps with other synucleinopathies; technical challenges in detection

**Table 5:** Genetic and Epigenetic Biomarkers.

Source Type	Biomarkers	Advantages	Limitations
Genetic	Specific gene mutations (APP, PSEN1, PSEN2, LRRK2, GBA)	Identify familial risk; help in early detection and understanding molecular mechanisms	Mostly relevant for familial cases; limited predictive value for sporadic disease
Genetic	Polymorphisms (e.g., APOE ε4)	Strong risk factor for sporadic Alzheimer’s; widely studied and validated	Does not guarantee disease development; influenced by environment and lifestyle
Epigenetic	DNA methylation changes, histone-modifying enzymes	Provide insight into gene regulation and disease progression; potential therapeutic targets	Detection requires specialized assays; dynamic and influenced by age, environment, and lifestyle

**Table 6:** Imaging Biomarkers.

Source Type	Biomarkers/Techniques	Advantages	Limitations
Imaging	Amyloid PET (e.g., Pittsburgh compound B, PiB)	Direct visualization of amyloid plaques; aids early diagnosis of Alzheimer’s	Expensive; limited availability; exposure to radioactive tracers
Imaging	Tau PET (e.g., flortaucipir)	Visualizes tau neurofibrillary tangles; tracks disease progression	High cost; limited accessibility; radiation exposure
Imaging	Structural and functional MRI	Non-invasive; assesses hippocampal atrophy, cortical loss, and network connectivity	May not be specific to a single pathology; interpretation requires expertise

**Table 7:** Comparison of Biological Fluid Biomarkers in Neurodegenerative Diseases.

Source Type	Biomarkers	Advantages	Limitations
**Cerebrospinal fluid**	Aβ42/Aβ40, t-tau, p-tau, α-syn	Direct from CNS; high accuracy	Invasive, risk of complications
**Blood**	p-tau217, NfL, GFAP	Non-invasive, repeatable, easy to collect	Concentration low; sensitivity issues
**Saliva**	α-syn, Aβ	Completely non-invasive, easy to collect	Very low concentration
**Urine**	Oxidative stress-related metabolites	Non-invasive, easy to collect	High variability

**Table 8:** Types of Biomarkers and Their Applications in Neurodegenerative Diseases.

Type of Biomarker	Examples of Biomarkers	Clinical Applications
**Protein**	Amyloid-beta, tau, alpha-synuclein	Diagnosis, monitoring disease progression, treatment evaluation
**Genetic**	Mutations in APP, PSEN1, PSEN2, HTT	Early diagnosis, risk assessment, genetic counseling
**Imaging**	Hippocampal volume, amyloid uptake in PET	Diagnosis, monitoring disease progression
**Body fluids**	Specific protein levels in CSF and blood	Diagnosis, prognosis, response to treatment
**Epigenetic**	DNA methylation patterns, histone modifications	Risk assessment, prognosis, response to treatment

**Table 9:** Types of Biomarkers in Intellectual Disabilities.

Biomarker Category	Down Syndrome	Fragile X Syndrome	Rett Syndrome
Part A: Molecular and Physiological Biomarkers			
**Genetic**	Trisomy of chromosome 21	Expansion of CGG repeats in FMR1 (>200 repeats)	Mutation in MECP2 gene
**Molecular**	Increased APP, increased Aβ42	Reduction or absence of FMRP (Fragile X Mental Retardation Protein)	Decreased BDNF, DNA methylation abnormalities
**Cerebrospinal fluid (CSF)**	Decreased Aβ42, increased phosphorylated tau	Increased MMP-9, altered neurotransmitter levels	Increased glutamate levels, disturbances in GABA levels
**Blood**	Increased SOD1, increased interleukin-6	Increased FMR1 mRNA, altered cytokine profile	Increased ammonium, metabolic profile changes
**Metabolic**	Folate metabolism abnormalities, increased oxidative stress	mGluR5 pathway dysfunction	Creatine metabolism abnormalities, increased lactate
**Inflammatory**	Increased TNF-α, IL-6, IL-1β	Increased IL-6, TNF-α, microglial dysfunction	Increased inflammatory cytokines, astrocyte activation
**Part B: Structural and Functional Biomarkers**			
**Imaging**	Cerebellar hypoplasia, reduced hippocampal volume	Enlarged brain ventricles, corpus callosum abnormalities	Reduced brain growth, microcephaly
**Electrophysiological**	Slow EEG waves, impaired event-related potentials	Increased sensitivity to sound, auditory processing deficits	EEG pattern with sharp and slow waves
**Neurodevelopmental**	Delayed myelination, reduced synaptogenesis	Impaired dendritic growth, reduced synaptic density	Reduced neuronal growth, impaired synapse formation

## Data Availability

No new data were created or analyzed in this study. Data sharing is not applicable to this article.
